# Extracellular Vesicles and Their Zeta Potential as Future Markers Associated with Nutrition and Molecular Biomarkers in Breast Cancer

**DOI:** 10.3390/ijms24076810

**Published:** 2023-04-06

**Authors:** Herminia Mendivil-Alvarado, Ana Teresa Limon-Miro, Elizabeth Carvajal-Millan, Jaime Lizardi-Mendoza, Araceli Mercado-Lara, Carlos D. Coronado-Alvarado, María L. Rascón-Durán, Iván Anduro-Corona, Daniel Talamás-Lara, Antonio Rascón-Careaga, Humberto Astiazarán-García

**Affiliations:** 1 Department of Nutrition, Research Center for Food and Development, CIAD, A.C., Hermosillo 83304, Mexico; herminia.mendivilal@gmail.com (H.M.-A.); limonmir@ualberta.ca (A.T.L.-M.); ecarvajal@ciad.mx (E.C.-M.); jalim@ciad.mx (J.L.-M.); coronado.carlosd@gmail.com (C.D.C.-A.); ivan.anduro@ciad.mx (I.A.-C.); 2 Department of Medicine, University of Alberta, Edmonton, AB T6G 2R7, Canada; 3 Undersecretariat of Prevention and Health Promotion, Secretary of Health of the Government of Mexico, Mexico City 11570, Mexico; araceli.mercado@salud.gob.mx; 4 Department of Chemical and Biological Sciences, University of Sonora, Hermosillo 83000, Mexico; lucila.rascon@unison.mx (M.L.R.-D.); antonio.rascon@unison.mx (A.R.-C.); 5 Department of Infectomics and Molecular Pathogenesis, Center for Research and Advanced Studies, IPN, Mexico City 14330, Mexico; daniel_talamas@hotmail.com

**Keywords:** nutritional status, extracellular communication, exosomes, microvesicles

## Abstract

A nutritional intervention promotes the loss of body and visceral fat while maintaining muscle mass in breast cancer patients. Extracellular vesicles (EVs) and their characteristics can be potential biomarkers of disease. Here, we explore the changes in the Zeta potential of EVs; the content of miRNA-30, miRNA-145, and miRNA-155; and their association with body composition and biomarkers of metabolic risk in breast cancer patients, before and 6 months after a nutritional intervention. Clinicopathological data (HER2neu, estrogen receptor, and Ki67), anthropometric and body composition data, and plasma samples were available from a previous study. Plasma EVs were isolated and characterized in 16 patients. The expression of miRNA-30, miRNA-145, and miRNA-155 was analyzed. The Zeta potential was associated with HER2neu (β = 2.1; *p* = 0.00), Ki67 (β = −1.39; *p* = 0.007), estrogen positive (β = 1.57; *p* = 0.01), weight (β = −0.09; *p* = 0.00), and visceral fat (β = 0.004; *p* = 0.00). miRNA-30 was associated with LDL (β = −0.012; *p* = 0.01) and HDL (β = −0.02; *p* = 0.05). miRNA-155 was associated with visceral fat (β = −0.0007; *p* = 0.05) and Ki67 (β = −0.47; *p* = 0.04). Our results reveal significant associations between the expression of miRNA-30 and miRNA-155 and the Zeta potential of the EVs with biomarkers of metabolic risk and disease prognosis in women with breast cancer; particularly, the Zeta potential of EVs can be a new biomarker sensitive to changes in the nutritional status and breast cancer progression.

## 1. Introduction

The lifestyle and food intake prior to the diagnosis of breast cancer influence the patients’ body composition; additionally, changes in body composition over the course of the disease can be a secondary effect of antineoplastic treatments [1,2].

The nutritional status and body composition are critical to cope with different conditions and treatments, including breast cancer [3]. Increased fat mass, loss of muscle mass, and/or bone mineral density can influence the disease prognosis in women diagnosed with breast cancer undergoing antineoplastic treatment [4,5,6]. Imbalances in body composition may increase the risk of toxicity from antineoplastic drugs, complications in surgical interventions, disease progression, and metastatic recurrence [7,8]. 

A pre-test post-test study conducted by our research group showed positive changes in the body composition of women recently diagnosed with breast cancer (*n* = 22) [9], where an individualized nutritional intervention was designed using the dynamic macronutrients meal-equivalent menu method [10]. In the above-mentioned study, the dietary plans were designed according to the clinical practice guidelines of the World Cancer Research Fund American Institute for Cancer Research [11] and the review of current evidence [12]. Resting energy expenditure was calculated with an equation designed in the Mexican population [13]. In patients with a BMI >25 kg/m^2^, a caloric restriction of 500–1000 kcal per day was made. The macronutrient distribution of the total caloric value was as follows: protein 1.2–1.5 g of protein per kilogram of weight, fat <30%, and carbohydrates 50%. Moreover, anthropometric and body composition analyses were performed on the patients at the beginning and 6 months after the nutritional intervention and antineoplastic treatment (weight, height, waist circumference, dual X-ray densitometry analysis [DXA], and electrical bioimpedance).

Results showed a significant loss of visceral fat, body weight, and no change in appendicular skeletal muscle mass, bone mineral density, and fat-free mass, after 6 months of nutritional intervention [9]. However, the changes in body composition discussed above can be detected after the first 6 months of antineoplastic treatment, when using conventional nutritional assessment tools and techniques. Further research into more sensitive techniques is needed at the molecular level, to better understand the molecular changes that occur in the early stages of disease development as well as in response to a nutritional intervention, instead of relying exclusively on traditional biomarkers for metabolic control and cancer progression. A biomarker is defined as a “characteristic that is measured as an indicator of normal biological processes, pathogenic processes, or biological responses to an exposure or intervention, including therapeutic interventions” [14]. Although biomarkers used in nutrition and cancer can detect changes in body composition, disease progression, the effect of a medication or antineoplastic treatment, etc., they are only sensitive at later stages of disease progression, and there is little to no specificity for nutritional changes [1,15].

The discrepancies between biomarkers used in clinical practice and research have been exposed, limiting the understanding of chronic diseases and nutrition [16]. Most biomarkers used in nutrition research (e.g., albumin, creatinine, glucose, etc.) are related to the development and progression of diseases. Still, there is a lack of biomarkers to assess early nutritional alterations or the response to nutritional interventions. This situation has driven the search for new biomarkers [2], among which extracellular vesicles (EVs) stand out.

In the last decade, EVs have been studied due to their active role in cell communication (see Mendivil et al., 2022 [17]). EVs are microparticles released by all cells into the extracellular space; their release and content respond to the conditions of the cellular microenvironment [18,19]. Using the classification of the International Society of Extracellular Vesicles, EVs are divided according to their size (nm) into small EVs and large EVs [19,20]. EVs are identified by specific markers that include but are not limited to CD63, CD81, ALIX, and TSG101 [19]. Additionally, the enrichment of proteins and markers in either the content or cytoplasmic and/or transmembrane proteins of EVs is highly varied. What determines the EVs content is still under investigation, but it is believed to depend on the nature of the cell where the EVs come from, as well as the conditions of the cellular microenvironment or the presence of diseases such as cancer.

As described above, EVs are sensitive to cellular stress, making EVs and changes in their characteristics (i.e., size, content, or Zeta potential) potential biomarkers in pathological conditions, ranging from neurodegenerative and chronic-degenerative diseases to breast cancer [21,22,23,24]. Among EVs characteristics, the Zeta potential is a biophysical property that explains the charges on the surface of a particle, resulting in electrostatic potential differences [25,26]. The Zeta potential gives EVs the ability to interact with other distal tissues and even with other EVs; however, little has been described about the changes in the Zeta potential for EVs throughout the course of diseases or support therapies (e.g., nutritional intervention). It is still unknown whether the use and validation of Zeta potential can be considered as a biomarker of the nutritional status and/or follow-up of nutritional interventions [27,28].

The nucleic acid content of EVs (i.e., miRNAs) has also been studied. miRNAs are short segments of RNA (~22 bases) that regulate gene expression through base pairing with complementary sequences of the target mRNAs [29]. Most of the detectable miRNAs in saliva and serum are concentrated in small EVs [30]. Therefore, miRNAs are transported from one tissue to another using EVs as a vehicle that provides protection from the transgressions of the extracellular environment. Specifically in breast cancer, miRNA-145, miRNA-30, and miRNA-155 have been identified as responsible for promoting angiogenesis, progression, and tumor invasion [31,32,33,34,35]; additionally, their expression has been associated with resistance to antineoplastic treatment and tumor recurrence [36]. Even when miRNA-145, miRNA-30, and miRNA-155 have been identified in breast cancer patients, where they play an active role in the breast cancer process, the information is limited regarding how these miRNAs that are being transported in EVs are involved in the progression of the disease or the development of comorbidities and metastases, as well as if they can be potential biomarkers for follow-up after a nutritional intervention.

Herein, we aim to explore the changes in the Zeta potential of EVs, as well as their miRNA-30, miRNA-145, and miRNA-155 content in plasma EVs of women recently diagnosed with breast cancer before and after 6 months of an individualized nutrition intervention and during active antineoplastic treatment.

## 2. Results

Herein, we show the analysis of EVs characteristics (the size, Zeta potential, and their content) in a subsample of women diagnosed with breast cancer, who participated in a quasi-experimental study in which they received a nutritional intervention during the first 6 months of antineoplastic treatment (see [9]). In this subsample, even when the number of volunteers decreased compared to the work by Limon-Miro et al. [9], the positive changes in body composition, such as decrease in total fat, visceral fat, and weight, were maintained.

Table 1 shows anthropometric and body composition characteristics in the sub-sample (*n* = 16) of women at baseline and after 6 months of a food-based nutritional intervention during active antineoplastic treatment.

Additionally, we analyzed the plasma concentration of biochemical biomarkers. At baseline, their median concentrations were glucose (114 mg/dL), triglycerides (109 mg/dL), triglyceride-glucose index (TyG index [8.6]), total cholesterol (206 mg/dL), HDL (52 mg/dL), VLDL (21 mg/dL), and LDL cholesterol (133 mg/dL). These values were not statistically different at the end of the intervention (*p* > 0.05).

**Figure 1:**
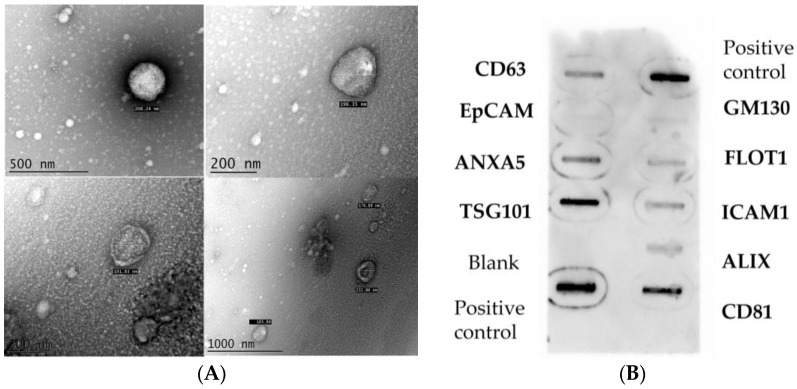
Morphology of the EVs through TEM and the expression of specific EVs markers. (**A**) The morphology and size of EVs using TEM; (**B**) Identification of specific EVs´ markers using the Exo-Check Exosome antibody Array™.

In this study, we isolated small- and large-sized EVs at baseline and 6 months after a food-based nutritional intervention. The median size of isolated EVs at baseline was 141 nm and 174 nm after 6 months (*p* > 0.05). Results show that the median Zeta potential was −8.8 mV at baseline and −8.0 mV at 6 months after the intervention (*p* > 0.05). When the morphology and size of the EVs were evaluated using transmission electron microscopy (TEM), spheroid shape vesicles with structures delimited by membranes were observed, consistent with the morphology of EVs. These EVs were dispersed and did not form aggregates; the approximate EV size was 200 nm (Figure 1A). Moreover, the expression of specific EV markers was confirmed (Figure 1B).

In this study, we also analyzed the EVs content for the expression of miRNA-145, miRNA-155, and miRNA-30 at baseline and after 6 months. The results showed a relative expression of 1.0 and 1.14 for miRNA-145; 0.72 and 0.84 for miRNA-155; and 1.0 and 0.93 for miRNA-30, before and after the nutritional intervention, respectively. However, the nutritional intervention had no impact on the normalized relative expression of the analyzed miRNAs (*p* > 0.05).

Results for the regression analyses for mixed effects models are summarized in Table 2 and Table 3. The outcome variables were the EVs diameter (nm), Zeta potential (mV), and their expression of miRNA-30, miRNA-145, and miRNA-155. As explanatory variables, we included anthropometric variables (weight and height), indexes (BMI, fat mass index, lean mass index, and triglyceride-glucose index [TyG index]), body composition (fat mass and visceral fat), physical activity (min/d), biochemical biomarkers (glucose, total cholesterol, LDL cholesterol, HDL cholesterol, and triglycerides), tumor molecular biomarkers (HER2neu, Ki67, and estrogen positive), and grade of breast cancer tumor (II, III). The EV size and miRNA-145 expression were not associated with any of the explanatory variables in the mixed effects models analysis (*p* > 0.05). The regression analyses for mixed effects models for the outcome variables miRNA-30, miRNA-155, and Zeta potential are summarized in Table 2.

Prior to the multivariate analysis, we performed a univariate analysis of the Zeta potential, selecting the following explanatory variables: HER2, Ki67, physical activity, weight, fat mass index, visceral fat, triglycerides, VLDL cholesterol, LDL cholesterol, and glucose (Table 2). To test our hypothesis on the influence of body composition on the characteristics of EVs, and in view of our previous results [37], we decided to include the variables weight, LDL cholesterol, and visceral fat in the multivariate analysis, despite their *p*-value limiting to 0.2. For the multivariate model, we excluded the fat mass index variable due to collinearity with two previously selected variables: weight and visceral fat (Table 2). In the end, the best variables that explained the Zeta potential of EVs were ER positive, HER2, Ki67, weight, and visceral fat (Table 3). The Zeta potential was negatively associated with Ki67 and weight, and positively associated with visceral fat, HER2, and ER positive (see Table 3).

On the other hand, for the univariate analysis of miRNA-155, the following variables were selected as explanatory variables: tumor molecular classification, ER positive, Ki67, weight, fat mass index, visceral fat, and HDL and LDL cholesterol (Table 2). We excluded the fat mass index variable, to avoid collinearity with weight and visceral fat. On the multivariate model, we decided to include the variables of molecular classification of the tumor, ER positive, and LDL cholesterol due to their previously reported association with the expression of miRNA-155 [38] and fat mass in patients with breast cancer; additionally, we included variables whose *p*-value was close to 0.2 (Table 2). The multivariate model showed that visceral fat (negative) and Ki67 (positive) were significant, and when combined, they formed the best association model (Table 3). Although the significance value for visceral fat is borderline at 0.05, it indicates a trend in its association with miRNA-155.

The selection of explanatory variables for the miRNA-30 were HER2, weight, visceral fat, total cholesterol, VLDL, LDL, and HDL (Table 2). On the multivariate analysis, we decided to exclude total cholesterol and include instead its components HDL, LDL, and VLDL, to be more specific with potential associations. For the multivariate analysis, we selected HDL and LDL cholesterol. We found a positive association between miRNA-30 expression and HDL cholesterol, and a negative association between LDL cholesterol (Table 3). Although HDL cholesterol has a level of marginal statistical significance (*p* = 0.053), we included it considering that the subsample of patients in our study is limited; thus, associations should be interpreted with caution until they are verified in a larger sample (Table 3).

## 3. Discussion

In this study, we isolated extracellular vesicles from the plasma of breast cancer patients and found EVs size to be classified as small and large. Changes in body composition of the women included in the sub-sample of the study are similar to that previously reported by our research group for the study population [9]. Despite the changes in body composition, there were no significant changes in traditional biomarkers such as glucose, insulin resistance, and serum lipids, at the beginning and end of the intervention.

In search of potential biomarkers that are sensitive to early nutritional changes, we found the characteristics of the EVs such as content, size, and their membrane Zeta potential, which to our knowledge has been poorly discussed in the literature.

### 3.1. Different Populations of Extracellular Vesicles

Extracellular vesicles have been proposed as cellular communication vehicles and as potential biomarkers for early disease diagnosis, treatment response, and prognostic monitoring [40,41,42]. Even though the use and validation of EVs are still being studied, the change in their characteristics (size, Zeta potential, or content) can provide information about the response that occurs at the cellular level to external aggressions, specific conditions such as therapeutic interventions [43,44,45,46], and potentially nutritional support therapies. Herein, we obtained small and large populations of EVs, but no significant difference in size was observed before and after the intervention, despite evidence suggesting that changes in EVs characteristics are associated with the nutritional status of the study subjects [47,48,49,50,51,52,53,54]. Most literature to date reports the use of the EVs size to classify them and do not use their size as a potential biomarker and its association with patient’s characteristics.

The changes observed in the characteristics of EVs in this work add to the limited evidence that exists to date on changes in the size and content of EVs in breast cancer, where it is suggested that EVs population size in cancers are mostly (but not limited to) small EVs [55]. Available literature is contrasting; on one hand, small EVs have been reported the most, and on the other, EVs larger than 1 µm [55] have been associated with progression of cancer and other processes related to the disease. EVs larger than 1 µm are suggested as large oncosomes, which belong to large EVs [19]; oncosomes have been characterized in the last decade as solely coming from tumor cells, so their content and membrane components have been related to tumor development and cancer progression [55,56,57]. However, in our results, we found EVs with sizes smaller than 1 µm; small EVs are also reported as potential carriers of key integrins in the development of metastases, such as the integrin ITGB3, which can favor communication between breast cancer cells [58]. In any case, the relation of EVs size should continue to be studied as an essential variable to better understand the process of progression and evaluation in cancers.

### 3.2. Associations between the Zeta Potential and Patient Characteristics

The EVs membrane is a highly interactive and dynamic surface area that is responsible for facilitating EVs interactions with the extracellular environment [59]. It has been suggested that the interactions of small EVs act as modulators of signaling in biological processes, such as metastasis, cancer regulation, and tissue regeneration [60]. Some of the mechanisms proposed to understand the interaction of EVs with other tissues are as follows: (1) fusion of the plasma membrane of EVs with the target cell, releasing its content inside; (2) vehicles of proteins and cellular components; upon arrival to the target cell, they can be fragmented by the action of proteases and act as a ligand for target cell receptors; and (3) endocytosis and pinocytosis [59,61]. Our results showed that the Zeta potential of EVs had several significant associations with clinical characteristics of the patients, as well as weight and visceral fat.

Based on our results, we hypothesize that the association of the Zeta potential and the transmembrane protein complex HER2 could be explained by a certain charge and membrane intensity that HER2 can exert over the EVs, contributing to its affinity for specific tissues. However, the evidence is limited [62], and more studies incorporating the Zeta potential within the characterization of EVs are needed. The presence of HER2 in the plasma EVs of patients with breast cancer has been reported [63]; additionally, the study conducted in 2016 by Sina et al. [64] showed that in small EVs from women diagnosed with breast cancer, 14 to 35% were enriched with HER2. Fei Tian et al. [65] suggest that the presence of proteins in the EVs membrane of tumor cells from different cell lines will confer a particular intensity percentage when using light scattering analysis; these properties will allow EVs to be differentiated among themselves, while potentially allowing their use in diagnostic, prognostic, and early detection of biomarkers, or in response to a specific pharmacological treatment [65,66]. Studies have shown that the presence of HER2 can be a promising biomarker in the identification of EVs whose origin is breast or prostate cancer [67]. Additionally, the heterogeneity in the components of EVs membrane from cancer, which can include HER2, has been associated with the overall survival of cancer patients [66]. To date, only a limited number of studies have tested the above results as predictive biomarkers of survival in cancer patients [68]; however, it is interesting to see how the understanding of the increasingly fine connections of EVs are being traced in their participation in cellular and molecular processes.

Our results showed that for the explanatory model of the Zeta potential, the variables included were visceral fat and weight. In studies of EVs and body composition, the EVs content was associated with the total and visceral fat mass [37,51,69,70]. Specifically, miRNAs linked to the development of comorbidities associated with excess weight have been identified in EVs from visceral adipose tissue; these microRNAs are linked to macrophage infiltration in adipose tissue, insulin resistance, and endothelial damage, among others [47,71]. Likewise, the size and composition of the EVs membrane have been positively associated with the amount of total body fat in overweight individuals [37,51,72,73]. However, to date, there is no evidence that links the amount of visceral fat with the Zeta potential of EVs in cancer. The Zeta potential of the EVs is determined by the amount and distribution of phospholipids, proteins, and carbohydrates of the membrane [74]. The positive association between visceral fat and the Zeta potential observed in our model could be associated with the loss of visceral fat that the women experienced in response to the intervention, in turn altering the composition of the EV membrane; unfortunately, there is no additional evidence to support our theory.

The Zeta potential determined in isolated EVs of women diagnosed with breast cancer was negative; this finding is similar to previous reports [40]. It is proposed that the negative charge can be a function of the content of carbohydrates, lipids, and proteins, as well as the side chains of its different ligands and receptors such as aspartate, lysine, arginine, and histidine, among others [40,75]. Additionally, EVs charge may be associated with transmembrane proteins such as tetraspanins, MHC class I (major histocompatibility complex), integrins, and GPI-anchored molecules (CD55 and CD59) that have been found in small EVs [20], as well as high levels of cholesterol, ceramides, and sphingomyelin [76,77]. In large EVs, its main components are phosphatidylserine, flotillin-1, and B1 integrins [78,79]. The components discussed above could coexist in the same group of EVs and confer a specific membrane charge.

The membrane composition and the Zeta potential of EVs will allow them to be part of different cell signaling pathways such as apoptosis, tumor growth, and proliferation, among others [48,80,81]. Some researchers have suggested that the protein content of EVs will depend on the cell of origin; for example, in breast cancer, Akagi et al. [82] suggest that depending on the cell line responsible for the disease, their load will be different and therefore communication in the cellular environment. However, it has also been proposed that the protein content of EVs can undergo post-transduction modifications such as glycosylation [46,77], which will expand their participation in different cellular signaling. Even the presence of glycans and glycoproteins have been studied as unique components of EVs originating from tumor cells, which would help to identify EVs in breast cancer and in subjects free of cancer [83]. In breast cancer, around 77 glycopeptides have been identified in plasma EVs from women with the disease, as well as changes in glycopeptides related to the type of cancer (metastatic and non-metastatic) [84], and its prognosis [85,86,87]. This suggests specific components of EVs as potential biomarkers for early diagnosis and response to treatment. However, validation studies are needed to corroborate the application of these as biomarkers. The identification of these components in EVs and their morphometric characteristics (Zeta potential and size) provide valuable information on molecular targets, which can be used in the development of targeted pharmacological therapies and nutritional interventions that help maintain the nutritional status of patients with breast cancer.

Although we found associations between the Zeta potential and biomarkers of cell proliferation and response to treatment such as Ki67 and estrogen receptor, to date, the evidence is scarce to try and understand to a better extent the involvement of EVs and the changes in their Zeta potential during processes such as cancer. Our hypothesis suggests that EVs change their Zeta potential, depending on the physiological context, such as metastasis, development, or progression of cancer. The change in the surface charge of the EVs membrane can potentially function as the first step in a signaling cascade that would include all the mechanisms by which the EVs can produce an effect, from protein transport or content release, to acting as a ligand of the same target cell. Even though promising information is known to date about the participation of EVs in different cellular processes in cancer, we are far from understanding the process of EV participation in detail. However, the study of EVs represents a research challenge; it is in constant development and our results add to the evidence and narrow the existing knowledge gap.

### 3.3. Relevance of miRNA Expression-Containing Plasma EVs in Breast Cancer Patients

The extracellular vesicles can carry and transport regulatory molecules such as oncogenic proteins, coding and non-coding RNAs, DNA, and lipids between neighboring cells and to distant sites. The presence of miRNAs has been identified in extracellular vesicles from patients with bone marrow, bladder, lung, liver, and breast cancers [88,89,90]. The contents of EVs have been proposed as potential biomarkers of the above diseases, especially as biomarkers of early risk and prognosis in different types of cancer [91,92]. Some of the most studied components as biomarkers of early risk in cancer are miRNAs, including miRNA-145, miRNA-155, and miRNA-30.

#### 3.3.1. Expression of miRNA-145 in EVs

The expression of miRNA-145 has been associated with tumor suppressor activity in breast cancer, inhibiting proliferation, migration, and invasion [93,94]; however, the suppressive activity and the mechanisms associated with the overexpression of miRNA-145 is still being studied. It has been suggested that tumor suppression by miRNA-145 is associated with the inhibition or stimulation of tumor angiogenesis; potential mechanisms of action are associated with the regulation of the insulin receptor substrate 1 (IRS1) gene, which suppresses N-RAS and VEGF-A (RAS mediates vascular endothelial growth factor), inhibiting tumor angiogenesis [32,95]. On the other hand, it has been suggested that tumor angiogenesis is promoted by reducing the expression of miRNA-145 in EVs from breast cancer cells [96]. The role of miRNA-145 has been highlighted in the development of metastasis and tumor proliferation in breast cancer cells by upregulating the expression of Transforming Growth Factor-beta Receptor 2 (TGFBR2) [97]. In addition, there is evidence that miRNA-145 could regulate tumor suppressor ZMYND10, because it can inhibit the miRNA-145 signaling pathway, thereby inhibiting the tumorigenicity of breast cancer [98].

On the other hand, the expression of miRNA-145 has been associated with key metabolic parameters in obesity and glucose metabolism, such as visceral fat area, HbA1c, plasma glucose, and circulating levels of leptin, adiponectin, and interleukin- 6 [99], as well as the risk of development cardiovascular disease (CVD).

Women with breast cancer have a higher risk of CVD and associated mortality, although the risk may vary depending on the history of antineoplastic treatment [100]. The risk of developing CVD is further increased in postmenopausal women [101]. Although our study subjects were free of cardiovascular diseases, some of them did have excess body weight [9], which can in turn increase the risk of developing CVD in adults with and without cancer [102]. In women with breast cancer, cardiovascular risk includes a series of factors ranging from eating habits, alcohol consumption, smoking, physical activity, and antineoplastic treatment. However, in breast cancer, the additional burden comes from the antineoplastic treatment, since it can cause hypertension, arrhythmias, valvular disease, and pericarditis, among other issues [103]. The novelty of our results adds to the limited evidence on the presence of miRNA-145 in EVs of breast cancer patients, and its use as a sensitive biomarker associated with CVD.

Mohasen et al. [94] reported the role of small EVs secreted from adipose tissue-derived mesenchymal stem cells (MSCs) on the transfection of miRNA-145 into breast cancer cells; they aimed to weaken their expansion and metastasis. Their results show that miRNA-145 can be considered as a potential therapeutic strategy in breast cancer. The presence of miRNA-145 in EVs has been documented in small EVs in several diseases including cancer, such as ovarian cancer [104], where miRNA-145 has even been suggested as the most promising biomarker for the preoperative diagnosis of ovarian cancer [105]. miRNA-145 has also been reported in urinary EVs from prostate cancer patients [106], in vascular smooth muscle cells [107], colorectal cancer [108], hepatocarcinoma [109], cerebral injury [110], as well as its active role in the recovery of skeletal muscle mass in mice with chemotherapy [111], osteoarthritis [112], and pancreatic cancer [113].

#### 3.3.2. Expression of miRNA-155 in EVs Was Associated with Ki67 and Visceral Fat

The presence of miRNA-155 in EVs from breast cancer has previously been reported [30,114,115]. Here, we showed the presence of miRNA-155 in EVs, but no significant difference was found between the initial and final expression; the limited sample size may have influenced the lack of power. The overexpression of miRNA-155 in metastatic breast cancer exosomes suggests its participation in the progression of breast cancer by suppressing the function of tumor suppressors such as PTEN and DUST14 [116,117]; the expression of miRNA-155 found in our study subjects may suggest that patients had a lower progression of breast cancer owing to downregulation, thereby promoting tumor growth and metastasis [118]. The underexpression of PTEN promotes the inhibition of Akt, a pathway that has already been reported to be deregulated in breast cancer [117,118]. Additionally, the expression of miRNA-155 has been previously reported and associated with resistance to antineoplastic treatment [36,119,120], tumor recurrence, and progression [119,121,122]. Likewise, the expression of miRNA-155 has been reported in EVs in diseases such as kidney cancer [123], lung injury [33], brain injury [124], and epilepsy [125], among others.

A low expression of miRNA-155 has been reported in patients under breast cancer treatment [126]. Stevic et al., reported a series of miRNAs contained in the EVs, associated with characteristics and clinicopathological parameters of breast cancer subtypes; coincidently, our results concur with their work, where they show an association in the expression of miRNA-155 with Ki67.

In breast cancer, the progression of the disease and the secondary effects of antineoplastic treatment promote the loss of muscle mass, reflected later in the development of cachexia [127,128]. The nutritional status of our study subjects improved post-intervention, but this might not have influenced the expression of miRNA-155. Evidence shows that the overexpression of miRNA-155 has been associated with a worsening of the nutritional status in patients with breast cancer [119]. It has been proposed that the development of alterations in the nutritional status of women with breast cancer is a function related to EVs, especially their content. In the case of miRNA-155, it has been suggested that EVs from breast cancer are emerging mediators of neoplastic cachexia [121], since they have an important role in the catabolism of adipocytes and muscle cells by targeting PPARγ (peroxisome proliferator-activated receptor gamma). In adipocytes, EVs from cancer cells contain miRNA-155, which promotes beige/brown differentiation and remodels metabolism in resident adipocytes by downregulating PPARγ expression but does not significantly affect biological conversion to C2C12 (myoblast cell line) [121]. Therefore, the transfer of miRNA-155 in EVs acts as an oncogenic signal that reprograms systemic energy metabolism and leads to cachexia associated with breast cancer [121,129].

In our results, we found the trend between visceral fat and expression of miRNA-155 of particular interest, because visceral fat was one of the body composition parameters that had a significant loss post-intervention. Thus, we hypothesize that this loss of visceral fat has an implication beyond anthropometric changes and extends to molecular changes such as miRNA-155 expression. This evidence provides the foundation for future studies to investigate miRNA-155 and its association with body composition in a larger cohort of patients. Although the expression of miRNA-155 in EVs in cancer has been related to the downregulation of PPARγ expression, it could also be associated with visceral fat [127]. PPARγ is essential for the differentiation and proliferation of adipocytes, and it has also a beneficial effect on inflammatory processes of the vascular wall [130,131]. In this sense, it has been reported that EVs from smooth muscle produce endothelial injury and promote atherosclerosis in endothelial cells [132,133]. Additionally, miRNA-155 has been associated with crosstalk between fibroblasts and macrophages, as well as in cardiac repair after an acute myocardial infarction [134]. Considering the current information on miRNA-155, we hypothesize that the relationship between the expression of miRNA-155 and visceral fat in EVs could be related to the development of cardiovascular diseases due to excess weight. However, research is lacking for validating our hypotheses.

Our results add to the current evidence and provide essential information on the expression of miRNAs contained in EVs in women with breast cancer undergoing antineoplastic treatment and a nutritional intervention. Even though the current evidence continues to expand the participation of this miRNA in the development of processes, the exact mechanisms and the role of the EVs in the transport, expression, or overexpression of this miRNA are still under study.

Although the analysis of miRNAs in EVs is promising as a diagnostic and prognostic biomarker of disease, it is not intended to replace the use of current biomarkers used in clinical practice. Rather, the expression of different miRNAs contained in the EVs reveal important information about the transport that these carry under conditions such as cancer. This information contributes to the understanding of the EVs in the body under different scenarios; in this case, that of patients with breast cancer who received a nutritional intervention.

#### 3.3.3. miRNA-30 Expression Was Associated with HDL and LDL Cholesterol

The expression of miRNA-30 has been related to different processes such as muscle depletion in situations of metabolic stress, muscular dystrophy [135,136], cardiovascular diseases [137], insulin resistance, diabetes [138], and breast cancer [35]. In our work, we found a negative association between LDL cholesterol and the expression of miRNA-30 and a positive association with HDL cholesterol. The association with metabolic biomarkers and miRNA-30 is still under study but the evidence from murine models suggests that this miRNA contained in EVs is a potential diagnostic biomarker for diabetes [138,139]. Likewise, in diabetes, miRNA-30 is attributed to active participation in the oxidation of fatty acids and endothelial dysfunction, suggesting it as a possible biomarker of coronary microvascular dysfunction [137,140], since the overexpression of miRNA-30 synergizes with exposure to fatty acids, thereby regulating eNOS (endothelial nitric oxide synthase) underexpression, a key regulator of microvascular function at the cardiac level [137].

In our results, the patients showed the expression of miRNA-30 in isolated EVs. A low expression of miRNA-30 in EVs has been associated with increased expression of genes involved in fibrosis and inflammation in ischemic remodeling, in murine and human models [141]. Herein, the expression of miRNA-30 was negatively associated with the level of LDL cholesterol and positively associated with HDL cholesterol, which may suggest an increased risk for the development of cardiovascular diseases in our study subjects. In murine models, miRNA-30, contained in EVs, has been proposed as a biomarker for the early diagnosis of cardiovascular disease, as well as a prognostic biomarker for tumor recurrence, improved survival, and metastasis in patients with breast cancer [142,143,144,145,146]. Additionally, miRNA-30 has been studied as a mediator in cancer invasion and migration by targeting KLF11 (krüppel-like factor 11), and activating the STAT3 pathway (Signal transducer and activator of transcription 3) [35]; these effects also play a role in different types of tumors, controlling critical signaling pathways and relevant oncogenes [147]. Some oncogenes have been associated with the expression of miRNA-30 and other miRNAs, as well as other breast cancer biomarkers such as HER2 [148].

The association of miRNA-30 with biomarkers of cardiovascular risk indicates a possible participation in the metabolism of LDL and HDL cholesterol; however, more studies are needed to help us understand the exact mechanisms by which this recently found effect occurs.

Although our sample size was a limitation, the models and associations were statistically significant. However, the associations we found should be taken with caution, since the presence of non-vesicular components will include but are not limited to lipoproteins owing to the isolation method we used. However, the associations we detected in our results support the continuation of the study of miRNA in EVs and their associations in future studies. In subsequent studies, our findings could be verified and/or expanded, but with a larger number of samples and considering our experimental limitations.

## 4. Materials and Methods

### 4.1. Subjects

Our work group developed and implemented a food-based nutrition intervention (6 months) in 22 recently diagnosed breast cancer patients undergoing antineoplastic treatment (for more information see [9,10]); here we worked with cryopreserved (−80 °C) plasma samples collected at baseline and after the intervention from a sub-sample (*n* = 16) of this study’s participants. The overall project was reviewed and approved by the ethics committee of the Center for Research in Food and Development (CIAD) (Code CE/05/2015). In accordance with the International Organizations of Medical Sciences, in bioethical guidelines 11 and 12 [149], we contacted the patients in the subsample who own the stored plasma and requested their authorization to carry out complementary plasma analyses: EVs size, Zeta potential, and content (miRNA-145, miRNA-155 and miRNA-30 content), as well as biochemical markers (i.e., glucose, total cholesterol, LDL cholesterol, HDL cholesterol, triglycerides, and insulin resistance).

Briefly, we will describe the previous study and variables used in the current study. The dietary intervention was based on the design of an individualized meal plan for each patient diagnosed with breast cancer [10]. Baseline and final assessments included body composition (visceral fat, total body fat, and fat-free mass) using dual X-ray absorptiometry (whole body mode; Discovery WI QDR SERIES. Hologic, Waltham, MI, USA) as well as physical activity and tumor molecular biomarkers (HER2neu, Ki67, and estrogen positive).

### 4.2. Isolation and Analysis of Extracellular Vesicles

Cryopreserved plasma samples were thawed at 4 °C and subsequently homogenized and centrifuged at 10,000× *g* for 30 min and then 3000× *g* for 15 min; subsequently, we performed the isolation and analysis of extracellular vesicle size, Zeta potential, and content (miRNA-145, miRNA-155, and miRNA-30). The isolation and analysis of EVs, as well as biochemical markers, were performed at baseline and 6 months after the intervention.

#### 4.2.1. Isolation of Plasma Extracellular Vesicles

EVs were isolated using ExoQuick^TM^ (System Biosciences, Cat. No. Exoq20a-1, Palo Alto, CA, USA) and the manufacturer’s protocol with additional centrifugation. We added 63 μL of ExoQuick^TM^ per 250 μL of plasma and left to precipitate overnight at 4 °C. After this time, the tubes were centrifuged at 1500× *g* for 30 min at 4 °C, discarding the supernatant and recovering the pellet. This was centrifuged again at 1500× *g* for 5 min at 4 °C, where the residues of the supernatant were carefully removed to maintain the EVs pellet.

The EVs pellet was processed and resuspended in 250 μL sterile 0.05X PBS for further analyses or storage. After isolation with ExoQuick^TM^, we used just the purification columns of the commercial kit ExoQuick^®^ ULTRA 125 (System Biosciences, Cat. No. EQULTRA-20A-1). Subsequently, we performed a protein quantification assay with the Micro BCA^TM^ Protein Assay Kit (Thermo Scientific^TM^ Product No. 23235, Waltham, MA, USA).

#### 4.2.2. Size and Zeta Potential Analyses

The size distribution and Zeta potential of EVs were analyzed using dynamic light scattering analysis (DLS) on a Möbiuζ (Wyatt Technology Corp., Santa Barbara, CA, USA) at 37 °C. From the resuspended EV pellet, we added 1500 μL of sterile 0.05X PBS, and then, it was loaded into disposable polystyrene cells for DLS. The size and Zeta potential were obtained using the software DYNAMICS 7.3.1.15 (Wyatt Technology Corp., Santa Barbara, CA, USA).

#### 4.2.3. Analysis of miRNA in Extracellular Vesicles

From the EVs pellet resuspended in PBS, total RNA extraction was performed using the miRNAeasy serum/plasma kit (Cat. No./ID: 217184, QIAGEN. Hilden, Germany). Subsequently, the miRNeasy Serum/Plasma Spike-In Control (Cat. No. # 219610, QIAGEN) was added. For cDNA synthesis, the TaqMan^®^ MicroRNA Reverse Transcription Kit (Applied Biosystems, Ref 4366596, Waltham, MA, USA) was used, as well as the specific primers for miRNA-145 (TaqMan™ MicroRNA Assay-Applied Biosystems, ID 002278) and miRNA-155 (TaqMan™ MicroRNA Assay-Applied Biosystems, ID 002623); for miRNA-30, the primer design was based on previous literature by Chen et al. [150] and Kramer et al. [151] and synthesized using Integrated DNA Technologies (see Supplementary Material: Table S1). Reverse transcription was performed in a T100 thermocycler (Bio-Rad. Serial # 621BR18873, Hercules, CA, USA), with the following conditions: 16 °C for 30 min, 42 °C for 30 min, and 85 °C for 5 min, at 4 °C.

The expression levels of miRNAs were quantified using the StepOne^TM^ Real-Time PCR (Applied Biosystems) and duplex PCRs were performed; we used a synthetic miRNA from Caenorhabditis elegans (cel-miR-39-3 p. TaqMan™ MicroRNA Assay-Applied Biosystems, ID 000200) as a reference control to normalize the data. The normalized expression of mature miRNAs was calculated for all samples, using miRNA-39 as the reference control gene. The Ct was defined as the PCR cycle in which the reporter dye fluorescence signal crosses an amplification threshold. Ct data were collected in the exponential phase of the PCR amplification [152]. The normalized relative expression was obtained with the ΔCt value of each target miRNA (145, 155 or 30); then, the relative quantity (RQ) with 2^^ΔCt^ was calculated for the target miRNA and reference (miRNA-39). Finally, the result was normalized via logarithmic transformation [153]. Log-transformed normalized expression was used for different statistical analyses.

#### 4.2.4. Negative Staining Electron Microscopy Analysis

The morphology and size of EVs were analyzed using transmission electron microscopy (TEM). The EVs pellet was fixed using 250 μL of glutaraldehyde 2.5% in 0.1 M Na^+^ cacodylate buffer (1:1); later, an aliquot was resuspended in 40 μL of sterile PBS filtered using 0.22 μm pore membranes, and subsequently adsorbed on carbon-coated copper grids with formvar mesh (0.3%) at room temperature. The grids were stained for 30 sec using a uranyl acetate solution (2.5%); the excess liquid was then removed. The samples were analyzed using the JEOL JEM-1011 transmission electron microscope (Jeol, Ltd., Tokyo, Japan).

#### 4.2.5. Specific Exosome Markers

Exosome quality was examined using Exo-Check Exosome Antibody Arrays (SBI system biosciences, EXORAY210B-8. Palo Alto, Santa Clara, CA, USA) following the manufacturer’s protocol. Exo-Check Exosome Antibody Arrays had a total of nine antibodies against eight known exosome markers (CD63, CD81, ALIX, FLOT1, ICAM1, EpCam, ANXA5, and TSG101) and one cis-Golgi marker (GM130).

#### 4.2.6. Analysis of Biochemical Biomarkers

We analyzed plasma glucose, total cholesterol, HDL cholesterol, LDL cholesterol, and triglycerides using the VITROS^®^ 250 System (Ortho Clinical Diagnostics, Raritan, NJ, USA). The triglyceride-glucose index (TyG index) was calculated using the following formula: [fasting triglycerides (mg/dL) × fasting plasma glucose (mg/dL)/2] [39].

#### 4.2.7. Statistical Methods

Regression analyses using mixed effects models were performed using the EV size, Zeta potential, and expression of miRNA-145, miRNA-155, and miRNA-30 as outcome variables. Anthropometric (body weight and height), body composition (visceral fat, total body fat, and fat-free mass), and tumor molecular biomarkers were included as explanatory variables. Regression coefficients and 95% confidence intervals were reported. *p* ≤ 0.05 was considered significant. Analysis was performed using STATA (v15.0 StataCorp LP, College Station, TX, USA).

Models were generated using a sequence of univariate and stepwise analyses. Univariate models were constructed and variables with a value of *p* ≤ 0.2 and biological plausibility were considered as possible explanatory variables, adjusted variables, or both [154,155]. These variables were considered for the stepwise model procedure. Variables with a *p*-value ≤ 0.05 were chosen for the final multivariate models along with their AIC (Akaike Information Criteria).

Differences between baseline and end-line expression of miRNAs, anthropometric variables, biochemical biomarkers, and EV characteristics were analyzed using Wilcoxon signed-rank test. Statistical analyses were performed using NCSS 2007 software.

## 5. Conclusions

Despite finding different EVs sizes in the plasma samples, we could not identify a significant difference before and after the nutritional intervention. However, the association of the Zeta potential (one of the characteristics of EVs) with different tumor biomarkers and body composition data stands out. Here, we showed that the characteristics of the EVs could contribute to explain different pathophysiological processes, as well as the development and progression of the disease. For its part, the expression of miRNA-30 and miRNA-155 contained in EVs was also influenced by changes in the levels of biomarkers of lipid metabolism. Our results add to the little evidence that exists on the miRNA-30, miRNA-155, and the Zeta potential, as a sensitive characteristic of EVs, so this characteristic should not be left aside in the study and search for new biomarkers in breast cancer. More evidence is needed for the use and validation of the Zeta potential and miRNAs in the clinical area.

An important limitation to our work was the sample size; however, our models show statistical significance between EVs characteristics and breast cancer. Characterizing the changes in the EVs under different stress situations will contribute to a better understanding of the development of the disease and to the development of new targeted therapeutic strategies, as well as the development of sensitive biomarkers of early risk, prognosis, or progression of the disease. More studies are required to elucidate these mechanisms.

## Figures and Tables

**Table 1:** Anthropometric and body composition characteristics of 16 women diagnosed with breast cancer at baseline and after 6 months food-based nutritional intervention during active antineoplastic treatment ^1^.

Variables	Nutritional Intervention		*p*-Value
	Baseline	6 Months	
Weight (kg)	72.5 (21.5)	69.6 (19.5)	0.002
Body mass index (kg/m^2^)	26.4 (8.1)	26.2 (7.3)	0.002
Fat mass (kg)	30 (16.9)	26.3 (16.2)	0.002
Fat mass (%)	41.6 (7.95)	39.3 (7.4)	0.03
Fat mass index (kg/m^2^)	11.1 (5.3)	10.1 (5.7)	0.002
Visceral fat (g)	697 (530.7)	590 (582.2)	0.01
Fat-free mass (kg)	40.3 (8.9)	41.1 (9.73)	0.08
Appendicular mass index (kg/m^2^)	5.1 (1.6)	5.7 (1.48)	0.06

^1^ Data are presented as median (IQR) interquartile range.

**Table 2:** Regression analyses using mixed effects models using miRNA-30, miRNA-155, and the Zeta potential as outcome variables in 16 women diagnosed with breast cancer.

Outcomes	miRNA-30				miRNA-155				Zeta Potential			
Explanatory Variables	Regression Coefficient	*p*-Value	95%CI		Regression Coefficient	*p*-Value	95%CI		Regression Coefficient	*p*-Value	95%CI	
TyG index ^1^	−0.375	0.344	−1.151	0.401	0.009	0.97	−0.493	0.512	0.231	0.706	−0.9696	−1.432
Glucose (mg/dL)	−0.006	0.394	−0.020	0.008	−0.0001	0.977	−0.009	0.008	−0.017	0.092 *	−0.038	0.002
VLDL (mg/dL)	−0.030	0.267 *	−0.084	0.023	−0.003	0.835	−0.036	0.029	0.057	0.125 *	−0.015	0.13
LDL (mg/dL)	−0.012	0.003 *	−0.020	−0.004	−0.004	0.204 **	−0.010	0.002	−0.008	0.239 **	−0.023	0.005
HDL (mg/dL)	0.024	0.121 *	−0.006	0.054	0.015	0.118 *	−0.003	0.033	−0.015	0.564	−0.066	0.036
Triglycerides (mg/dL)	−0.001	0.597	−0.009	0.005	0.0008	0.708	−0.003	0.005	0.007	0.124 *	−0.002	0.017
Total cholesterol (mg/dL)	−0.009	0.021 *	−0.016	−0.001	−0.002	0.461	−0.007	0.003	−0.005	0.374	−0.018	0.007
Lean mass index (kg/m^2^)	−0.094	0.576	−0.428	0.238	−0.103	0.326	−0.310	0.103	0.013	0.957	−0.492	0.520
Visceral fat (g)	−0.0006	0.289 *	−0.001	0.0005	−0.0007	0.087 *	−0.001	0.0001	0.001	0.21 **	−0.0007	0.003
Fat mass index (kg/m^2^)	−0.028	0.698	−0.171	0.114	−0.081	0.062 *	−0.167	0.004	0.235	0.029 *	0.024	0.446
Physical activity (minutes)	−0.0001	0.898	−0.002	0.002	0.0001	0.84	−0.001	0.001	−0.002	0.192 *	−0.005	0.001
Weight (kg)	−0.015	0.298 *	−0.043	0.013	−0.017	0.05 *	−0.035	0.00003	0.026	0.291 **	−0.022	0.075
Ki67	0.408	0.334	−0.419	1.235	0.462	0.086 *	−0.065	0.989	−0.937	0.199 *	−2.368	0.493
ER-positive	−0.004	0.993	−0.966	0.958	−0.378	0.242 **	−1.013	0.255	1.643	0.032 *	0.138	3.147
HER 2/NEU (+)	0.852	0.048 *	0.006	1.698	0.066	0.834	−0.553	0.686	1.374	0.063 *	−0.075	2.825
Luminal A	−0.515	0.575	−2.315	1.284	−0.292	0.63	−1.486	0.900	−1.206	0.456	−4.383	1.969
Luminal B	−0.808	0.384	−2.626	1.010	−0.707	0.25 **	−1.912	0.498	−0.128	0.937	−3.338	3.080
BC Grade II	0.102	0.876	−1.191	1.397	−0.077	0.862	−0.950	0.795	−0.947	0.423	−3.266	1.370
BC Grade III	0.124	0.886	−1.570	1.819	0.412	0.479	−0.730	1.555	−1.082	0.485	−4.118	1.953

^1^, [fasting triglycerides (mg/dL) × fasting plasma glucose (mg/dL)/2] [39]. ER-positive, estrogen positive; HER2/NEU(+), human epidermal growth factor 2; HDL, high-density lipoprotein; LDL, low-density lipoprotein; VLDL, very-low-density lipoprotein; 95%CI, confidence intervals; MC, molecular classification of the tumor. BC, breast cancer grade. * When *p* ≤ 0.2 in the univariate analysis, the variables were considered for the stepwise multivariate analysis. ** Variables selected by suggestion of the conceptual framework and they were of interest to the research group.

**Table 3:** Mixed model effects analysis for miRNA-30, miRNA-155 expression, and the Zeta potential in 16 women diagnosed with breast cancer.

Outcomes	Explanatory Variables	Regression Coefficient	*p*-Value	95%CI		AIC
miRNA-155	Visceral fat (g)	−0.0007	0.050	−0.001	−7.04 × 10^−7^	87.731
	Ki67	0.478	0.049	0.002	0.953	
Zeta potential	ER-positive	1.572	0.010	0.370	2.775	132.326
	HER2/NEU(+)	2.159	0.000	1.111	3.206	
	Ki67	−1.391	0.007	−2.401	−0.381	
	Weight (kg)	−0.094	0.005	−0.160	−0.028	
	Visceral fat (g)	0.004	0.000	0.002	0.0075	
miRNA-30	HDL (mg/dL)	0.026	0.053	−0.0003	0.053	116.137
	LDL (mg/dL)	−0.012	0.001	−0.020	−0.004	

AIC, Akaike Information Criteria; 95%CI: confidence intervals; ER-positive, estrogen positive; HER2/NEU(+), human epidermal growth factor 2; HDL, high-density lipoprotein; LDL, low-density lipoprotein.

## Data Availability

Not applicable.

## Supplementary Materials

The following supporting information can be downloaded at: https://www.mdpi.com/article/10.3390/ijms24076810/s1.
