# Supplementary material for: Extracellular Vesicles and Their Zeta Potential as Future Markers Associated with Nutrition and Molecular Biomarkers in Breast Cancer
Source: Int J Mol Sci. 2023 Apr 6;24(7):6810. doi: 10.3390/ijms24076810 (PMC10094966; doi:10.3390/ijms24076810)
Supplement: Supplementary file 1 [file ijms-24-06810-s001.zip › ijms-2265762-supplementary.pdf]

Table S1. Design of miRNA-30 primers

**Sequence –** FORWARD QPCR PRIMER	25nmole DNA Oligo, 21 bases
**5´- CAC GCA TGT AAA CAT CCC CGA – 3´**	
**Properties**	**Amount of Oligo**
Tm (50mM NaCl): 57.7 °C	4.7= 23.1 =0.15
GC Content: 52.4%	OD_{260} nmoles mg
Molecular weight: 6344.2	
nmoles/OD260: 4.9	For 100 μM: add 231μL
ug/OD260: 31.1	
Ext. Coefficient: 202,800 L/(mole·cm)	
**Secondary structure calculations**	
Lowest folding free energy (kcal/mole): -0.22 at 25°C	
Strongest folding *Tm*: 28.1 °C	
**Sequence –** REVERSE QPCR PRIMER	25nmole DNA Oligo, 20 bases
**5´-CCA GTG CAG GGT CCG AGG TA– 3´**	
**Properties**	**Amount of Oligo**
Tm (50mM NaCl): 61.3 °C	4.7= 21.4 =0.13
GC Content: 65%	OD_{260} nmoles mg
Molecular weight: 6,183	
nmoles/OD260: 5.1	For 100 μM: add 214μL
ug/OD260: 31.4	
Ext. Coefficient: 196,900 L/(mole·cm)	
**Secondary structure calculations**	
Lowest folding free energy (kcal/mole): 0.61 at 25°C	
Strongest folding *Tm*: 13 °C	
**Sequence –** OLIGO DE RT	25nmole DNA Oligo, 50 bases
**Properties**	**Amount of Oligo**
Tm (50mM NaCl): 71 °C	10= 21.1 =0.32
GC Content: 56%	OD_{260} nmoles mg
Molecular weight: 153,378	
nmoles/OD260: 2.1	For 100 μM: add 211μL
ug/OD260: 32.3	
Ext. Coefficient: 476,500 L/(mole·cm)	
**Secondary structure calculations**	
Lowest folding free energy (kcal/mole): -14.50 at 25°C	
Strongest folding *Tm*: 68.6 °C	
**Sequence –**SONDA MIR30D	100 nm PrimeTime MGB Probe 5´6-FAM/ 3´MGB-NFQ, 17 bases
**5´- /56-FAM/CTG GAT ACG ACC TTC CA/3MGB-NFQ/– 3´**	
**Properties**	**Amount of Oligo**
GC Content: 52.9%	
Molecular weight: 6,788.1	100 μM in 232μL IDTE Buffer pH 8.0
nmoles/OD260: 4.3	
ug/OD260: 29.1	
Ext. Coefficient: 233,360 L/(mole·cm)	
**Secondary structure calculations**	
Lowest folding free energy (kcal/mole): -0.80 at 25°C	
Strongest folding *Tm*: 33.3 °C	
Secondary structure should not affect yield or purity for this oligo.	
